# Supplementary material for: Vena Cava Thrombus in Patients with Wilms Tumor
Source: Cancers (Basel). 2022 Aug 14;14(16):3924. doi: 10.3390/cancers14163924 (PMC9405781; doi:10.3390/cancers14163924)
Supplement: Supplementary file 1 [file cancers-14-03924-s001.zip › cancers-1871386-supplementary.pdf]

PSupplementary Materials

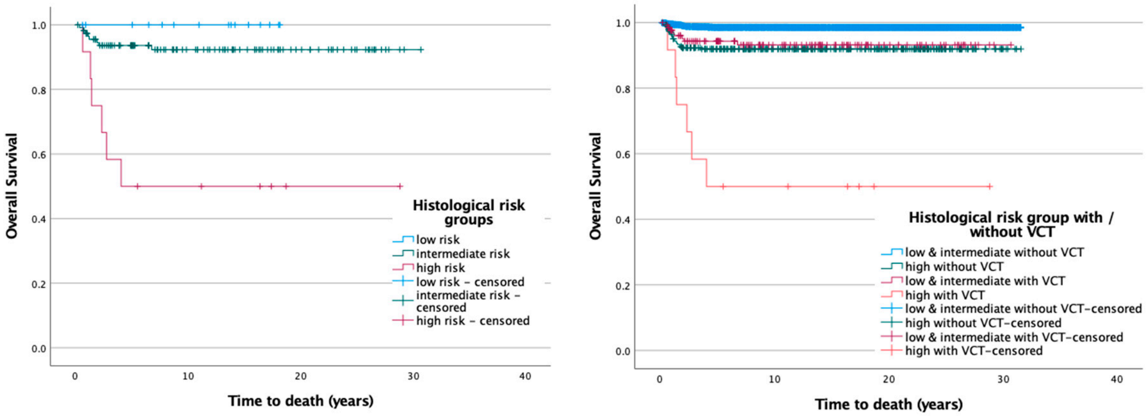

(**a**) (**b**)

Figure S1. (**a**) OS by histological risk group in patients with VCT (low risk 100%, intermediate risk 93%, high risk 50%, log rank $p < 0.001$); (**b**) OS depending on histological risk group and thrombus in patients with and without VCT (low and intermediate risk without VCT 98.6%, high risk without VCT 92.3%, low and intermediate risk with VCT 93.8, high risk with VCT 50%, log rank $p < 0.001$).

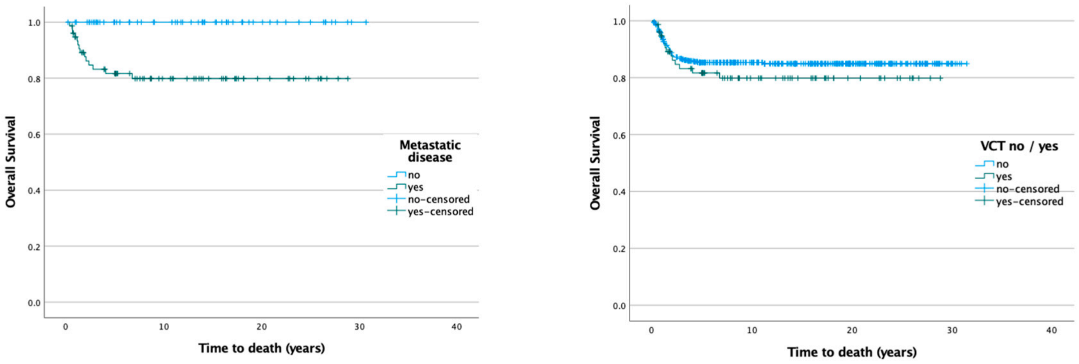

(**a**) (**b**)

Figure S2. (**a**) OS of patients with VCT in relation to the presence of metastases at time of diagnosis (no 100%, yes 81.8%, log rank $p < 0.001$); (**b**) OS of patients with metastatic disease at diagnosis with and without VCT (with VCT 81.8%, without VCT 85.7%, log rank $p = 0.341$).

Table S1. Baseline data of the entire cohort categorized into patients with and without VCT. Median with interquartile ranges (25%/75%), * = chi^2-test, # = Mann–Whitney U-test.

**Characteristics**		**Vena Cava Thrombosis**		***p***
		**Controls**	**Cases**	
Sex	Male	1346	62	0.238*
	Female	1519	86	
Affected side	Right	1269	83	0.059*
	Left	1338	57	
	Both sides	204	8	
	Extrarenal	12	-	
Age	Median (months)	39 (21/62)	56 (35/74)	**<0.001#**
	0–48 months	1592	62	**<0.001***
	> 48 months	1058	80	
Primary therapy	Surgery	329	5	**0.002***
	Chemotherapy	2460	142	
Tumor volume	< 500 ml	1633	69	**0.004***
	>= 500 ml	1031	72	
Histology	Low risk	92	17	**<0.001***
	Intermediate risk	2333	119	
	High risk	442	12	
Pathological stage	I	1732	18	**<0.001***
	II	618	54	
	III	502	75	
Preoperative rupture of tumor capsule	No	2749	141	0.715*
	Yes	118	7	
Appearance of renal vein	Normal	2527	22	**<0.001***
	Pathological	87	17	
	Infiltrated by tumor	38	10	
	Thrombosis	53	94	
Appearance of regional lymph nodes	Normal	1606	69	**0.013***
	Pathological	947	62	
	Infiltrated by tumor	66	7	
Metastases at diagnosis	No	2387	64	**<0.001***
	Yes	436	81	
Lung	No	2462	70	**<0.001***
	Yes	405	78	
Liver	No	2813	126	**<0.001***
	Yes	54	22	
Extraabdominal	No	2853	142	**<0.001***
	Yes	14	6	
Mediastinum	No	2857	145	**0.002***
	Yes	10	3	
Abdominal	No	2843	146	0.509*
	Yes	24	2	
Bone	No	2854	145	**0.010***
	Yes	13	3	
Soft tissue	No	2865	147	**0.023***
	Yes	2	1	
Brain	No	2891	124	-
	Yes	-	-	

Table S2. Change of thrombus due to chemotherapy (*n* = 118) according to histology (*p* = 0.014) and risk classes (*p* = 0.034).

Histology	No Change		Change		Unknown		No Change		Change		Unknown		Risk Class
	n	%	*n*	%	*n*	%	*n*	%	*n*	%	*n*	%	
Completely necrotic type	12	70.6	4 ↓, 1 ↑	29.4	-	-	12	70.6	4 ↓, 1 ↑	29.4	-	-	Low risk
Epithelial type	5	83.3	1 ↓	16.7	-	-							
Stromal type	8	61.5	2 ↓, 2 ↑	30.8	1	7.7							
Mixed type	14	87.4	1 ↓	6.3	1	6.3	67	73.6	20 ↓, 2 ↑	24.2	2	2.2	Intermediate risk
Regressive type	38	70.4	16 ↓	29.6	-	-							
Focal anaplasia	2	100	0 ↓	-	-	-							
Blastemal type after preoperative chemotherapy	3	60	2 ↓	40	-	-	7	70	3	30	-	-	High risk
Diffuse anaplasia	4	80	1 ↑	20	-	-							
All	**86**	**72.9**	**30**	**25.4**	**2**	**1.7**	**86**	**72.9**	**30**	**25.4**	**2**	**1.7**	All
